# 1-Deoxynojirimycin: Occurrence, Extraction, Chemistry, Oral Pharmacokinetics, Biological Activities and In Silico Target Fishing

**DOI:** 10.3390/molecules21111600

**Published:** 2016-11-23

**Authors:** Kuo Gao, Chenglong Zheng, Tong Wang, Huihui Zhao, Juan Wang, Zhiyong Wang, Xing Zhai, Zijun Jia, Jianxin Chen, Yingwu Zhou, Wei Wang

**Affiliations:** 1 Beijing University of Chinese Medicine, Bei San Huan East Road, Beijing 100029, China; linfengtingchan@foxmail.com (K.G.); zheng1633@163.com (C.Z.); hh686@126.com (H.Z.); doctorjuanwang@163.com (J.W.); bucmwzy@126.com (Z.W.); zhaixing_918@163.com (X.Z.); 15652605582@163.com (Z.J.); cjx@bucm.edu.cn (J.C.); 2 Beijing Gulou Hospital of Traditional Chinese Medicine, 13 DouFuChi Hutong, Dongcheng District, Beijing 100009, China; selenss@163.com

**Keywords:** iminosugar, mulberry, *Bacillus*, fermentation, silkworms, antihyperglycemic, anti-obesity, antiviral, molecular targets

## Abstract

1-Deoxynojirimycin (DNJ, C_6_H_13_NO_4_, 163.17 g/mol), an alkaloid azasugar or iminosugar, is a biologically active natural compound that exists in mulberry leaves and *Commelina communis* (dayflower) as well as from several bacterial strains such as *Bacillus* and *Streptomyces* species. Deoxynojirimycin possesses antihyperglycemic, anti-obesity, and antiviral features. Therefore, the aim of this detailed review article is to summarize the existing knowledge on occurrence, extraction, purification, determination, chemistry, and bioactivities of DNJ, so that researchers may use it to explore future perspectives of research on DNJ. Moreover, possible molecular targets of DNJ will also be investigated using suitable in silico approach.

## 1. Introduction

Polyhydroxylated piperidines and their derivatives are famous for their excellent bioactivities [1]. Iminosugars contain an analog of pyranose ring or d-glucose, in which the ring oxygen atom is replaced by a nitrogen atom. 1-Deoxyiminosugars are chemically more stable than normal iminosugars because of absence of a hydroxyl group at the C1 position. Among the iminosugars, naturally occurring 1-deoxyiminosugars such as DNJ are strong glycosidase inhibitors [2]. Originally, reduction of nojirimycin led to the chemical synthesis of DNJ [3]; afterward, DNJ was found from natural source, i.e., mulberry tree root and *Bacillus* species [4,5].

**Figure 1:**
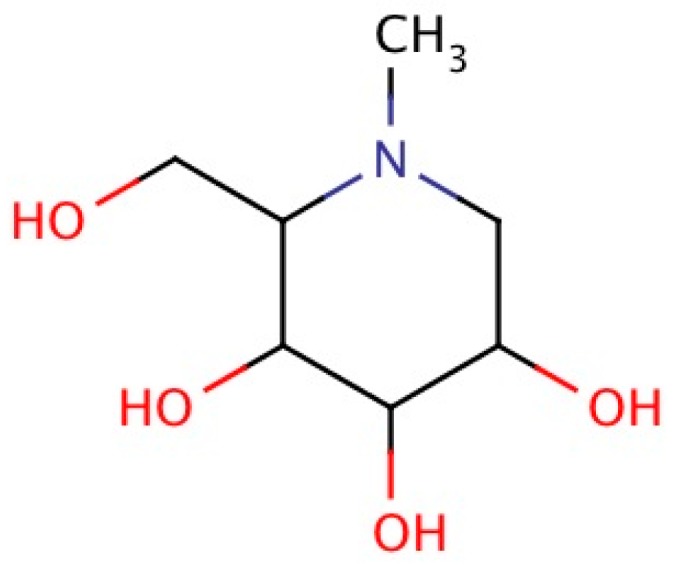
Chemical structure of 1-Deoxynojirimycin [7].

1-Deoxynojirimycin (DNJ, C_6_H_13_NO_4_, 163.17 g/mol) (Figure 1), an alkaloid azasugar or iminosugar, is a biologically active natural compound [6,7,8]. The IUPAC name of DNJ is (2*R*,3*R*,4*R*,5*S*)-2-(hydroxymethyl)piperidine-3,4,5-triol. It is also known as moranoline [9]. Various derivatives of DNJ are *N*-azidopropyl-1-deoxynojirimycin, *N*-nonyl-1-deoxynojirimycin, 1-deoxynojirimycin-6-phosphate, and *N*-methyl-1-deoxynojiri-mycin-6-phosphate [8]. It exists in mulberry leaves and *Commelina communis* (dayflower) as well as from several bacterial strains such as *Bacillus* and *Streptomyces* species [7].

After extraction, DNJ contents can be determined by HPLC using various detectors such as fluorescent, evaporative light-scattering detector, pulsed amperometric, and Mass spectrometer [10,11,12]. Since DNJ molecule does not contain chromophore, the quantification of DNJ through spectral analysis needs derivatization, i.e., an analytical approach for quantification of nitrogen-containing compounds [13,14,15,16]. To quantify and resolve structural information of DNJ by GC-MS, Bajpai and Rao used trimethylsilyl (TMS) derivatization [7].

The pharmacokinetic of DNJ after oral administration has been studies by some researchers [17,18,19]. Moreover, DNJ has many biological activities, including antihyperglycemic [20,21,22,23,24,25], anti-obesity [26,27,28,29,30], and anti-viral [31] have also been reported [32,33,34,35].

Currently, the prediction of biological targets of small drug molecules has become very easy owing to the rapid growth of bioactivity databases, in silico target fishing approaches and accessible web services [36]. There are many approaches to in silico target fishing including the structure-based and the ligand-based methods, data mining, and chemical similarity searching [37]. Then, the output data of these approaches are validated by adopting useful modality. The structure-based target prediction methods are appropriate to the drug-like small organic entities, which induce biological effects but have ambiguous macromolecular targets [36].

Therefore, the aim of this detailed review article is to summarize the existing knowledge on occurrence, extraction, purification, determination, chemistry, and bioactivities of DNJ, so that the researchers may use it to explore future perspectives of research on DNJ. Moreover, possible molecular targets of DNJ will also be investigated using suitable in silico approach.

## 2. 1-Deoxynojirimycin

### 2.1. Occurrence

**Figure 2:**
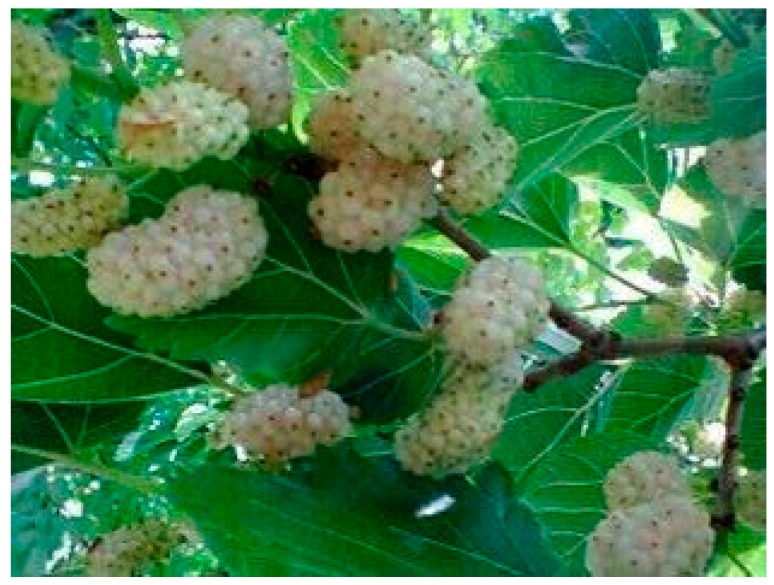
Mulberry leaf and fruit.

**Figure 3:**
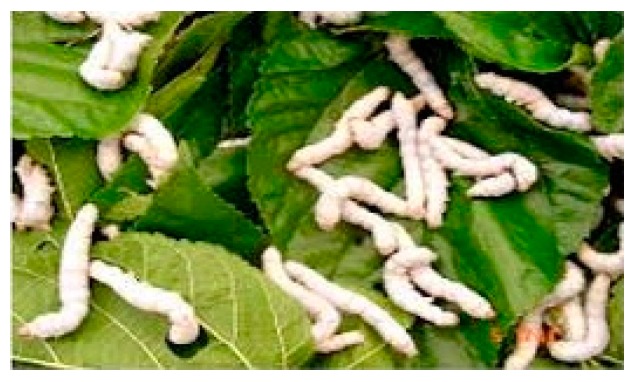
Silkworm larvae consuming mulberry leaves.

1-Deoxynojirimycin (DNJ), a product of fermentation, exists in mulberry leaves and *Commelina communis* (dayflower) as well as is isolated from several bacterial strains such as *Bacillus* and *Streptomyces* species [7,8]. Mulberry, a common deciduous plant, belongs to the genus *Morus* (Moraceae family). The botanical and pharmaceutical names of mulberry tree are *Morus alba* L. and *Folium mori*, respectively [38]. Mulberry tree is found in countries with a subtropical or mild temperate environment, including China, Japan, Korea, India, Pakistan, and other Asian countries [39]. By reason of folklore tonic, mulberry leaves have been used as an anti-diabetic tea. The general use of mulberry includes silkworm (*Bombyx mori* L.) feeding (Figure 2 and Figure 3) [40], fruit production [41], and medicine preparation [42,43]. Mulberry leaves are used as a source of protein in food products [44]. Many scientific studies have reported the medicinal importance of mulberry [45]. Mulberry leaves are traditionally used as medicine for controlling blood sugar level [20,46,47,48]. Studies have also reported the effectiveness of mulberry leaves in skin aging [49,50] and neurodegenerative disorders including Alzheimer’s disease and Parkinson’s disease [51]. Moreover, sedative effect of mulberry fruits and anti-inflammatory, diuretic, antitussive, and antipyretic properties of mulberry root bark have been studies [52]. In addition to DNJ, many other bioactive compounds including flavonoids, alkaloids, steroids, and coumarins also exist in mulberry leaves [53]. DNJ constitutes only 0.11% (*w*/*w*) of mulberry leaf [20,48], while the synthesis of DNJ is a complex process [54]. For this reason, DNJ is an expensive compound. Thus, the possible intense demand for DNJ in future has urged exploring other sources of DNJ, including various bacterial strains such as *Bacillus* [7], *Streptomyces* [8], *Actinoplanes* [55], and *Flavobacterium saccharophilium* species [56].

Numerous researchers have reported the DJN contents in different parts and various varieties of mulberry [25,57,58,59,60]. Various mulberry varieties contain DNJ contents ranging between 0.68 and 2.78 mg/gm [7], while this fraction varies between 1.57 and 3.48 mg/gm in different Chinese mulberry leaves [25]. Moreover, mulberry shoots contain the highest contents of DNJ, followed by young mulberry leaves. Mature leaves of mulberry contain the least contents DNJ [6]. Another group of investigators has reported the quantity of DJN contents in mulberry leaves fermented by different microorganisms, such as *Lactobacillus plantarum* (*lactic acid bacteria*), *Zygosaccharomyces rouxii* (*yeast*), *Wickerhamomyces anomalus* (*yeast*), and *Bacillus subtilis*. They also examined the extent to which the fermented mulberry leaf powder extract (FMLE) inhibited the α-glucosidase activity. All the mulberry leave groups showed 1–2-fold increase in DNJ contents in comparison to unfermented mulberry leaf powder extract (UFMLE). Additionally, FMLE exhibited higher α-glucosidase activity compared to UFMLE [61]. Resultantly, DNJ-rich food products can be prepared by fermenting mulberry leaves using the above-mentioned fermenting agents.

### 2.2. Extraction

There are four routes to produce DNJ: (i) extraction from plants such as the mulberry trees; (ii) extraction from silkworm; (iii) chemical synthesis following different synthetic strategies; and (iv) fermentation by various *Bacillus* or *Streptomyces*. Currently, the use of mulberry dry tea is flourishing as functional food. The content of DNJ is as low as approximately 100 mg/100 g of dry tea. This fraction of DNJ is biological ineffective (Biologically effective dose of DNJ is 6 mg per 60 kg human weight). Thus, the possible intense demand for DNJ in future has urged exploring new strategies for its efficient extraction and purification. In this context, a modality was proposed by Ezure et al. who developed a rapid screening approach (oblate agar plate method) for isolation of DNJ-producing *Streptomyces lavendulae* GC-148. Its DNJ spectra were identical to that obtained from mulberry [9]. Afterwards, they noted 27–33 folds increase in the production of DNJ through media improvement and mutagenic treatments (ultraviolet irradiation and *N*-methyl-*N*′-nitro-*N*-nitrosoguanidine treatment). Furthermore, to develop tea with higher DNJ content, Vichasilp et al. studied 35 Thai mulberry varieties to explore the content distribution and α-glucosidase inhibitory activity of DNJ [6]. DNJ content among various mulberry varieties ranged between 30 and 170 mg/100 g of dry leaves. They found that the mulberry shoots contained the highest contents of DNJ (300 mg of DNJ/100 g of dry shoot), followed by young mulberry leaves. Mature leaves of mulberry contained the least contents DNJ [6]. Thus, the shoot is the most suitable part of mulberry tree for the preparation of biological effective tea for the suppression of postprandial blood glucose. A current study has stated the use of a statistical procedure, response surface methodology (RSM), for the optimization of extraction efficiency of DNJ. The optimum extraction conditions at which DNJ yield was maximum (256 mg of DNJ per 100 g of dry mulberry leaves) is given here: ethanol concentration of 55%, extraction temperature of 80 °C, extraction time of 1.2 h and ratio of solvent to sample of 12:1. For efficient separation of DNJ from other components in mulberry leaves extracts, a column packed with a selected 732 resin was used. The recovery and purity of DNJ in the end product were >90% and >15%, respectively [62]. Jiang et al. stated that fermentation of mulberry leaf by *Ganoderma lucidum* produced the highest DNJ content [52]. The optimal condition for mulberry fermentation, obtained from RSM, was the following: pH 6.97, potassium nitrate content 0.81%, and inoculums volume 2 mL. The recovery of DNJ in the end product was 2.74 fold of those in mulberry leaf [52]. Another study described the use of Ultrasound-assisted extraction technique for mulberry DNJ extraction. The extraction efficiency and productivity of DNJ in the end product were 98% and 20%, respectively [63].

### 2.3. Quantitation of DNJ

It is difficult to quantify DNJ using ultraviolet or fluorescence detector because 1-deoxynojirimycin is a polar compound lacking a chromophore. Moreover, it is not retained or quantified by generally used reverse-phase chromatography columns. Instead, DNJ is partially retained or quantified using ligand-exchange and aminopropyl columns [10]. However, many other methods have been developed for quantifying DNJ. For example, hydrophilic interaction liquid chromatographic (HILIC) method using an evaporative light-scattering detector (ELSD) has been developed for determining water-soluble compounds such as DNJ [11]. The HILIC column has shown considerable retention potential of hydrophilic compounds; rather the compounds containing amino groups, the iminosugars such as DNJ, have exhibited good retention in HILIC-ELSD system [64]. Another study has reported the use of 9-fluorenylmethoxycarbonyl chloride for determining DNJ using reverse-phase high-performance liquid chromatography (HPLC) coupled with a fluorescence detector. This method works on the basis of secondary amino groups in DNJ [65]. Besides, many other methods, including HPLC-MS/MS [66], HILIC-MS/MS [67], and high-performance anion-exchange chromatography using pulsed amperometric detector (HPAEC-PAD) [12], have been developed for determining DNJ concentration. Using HPLC-MS/MS, Nuengchamnong et al. reported the isolation of DNJ from the mulberry leaf extract on a TSK gel Amide-80 column using a mobile phase mixture of 0.1% formic acid and acetonitrile. The limits of detection and quantitation were 100 pg and 75 pg, respectively [66].

Since DNJ molecule does not contain chromophore, the quantification of DNJ through spectral analysis needs derivatization, i.e., an analytical approach for quantification of nitrogen-containing compounds [13,14,15,16]. To quantify and resolve structural information of DNJ by GC-MS, Bajpai and Rao used trimethylsilyl (TMS) derivatization [7]. The disadvantage of this derivatization is the requirement of water removal from samples for silylation [7,65,67]. Another study has reported the derivatization of mulberry sample by using 9-fluorenylmethyl chloroformate, which reacts with primary and secondary amines under mild conditions producing DNJ derivatives. However, the reaction of 9-fluorenylmethyl chloroformate with tertiary amines requires dealkylation [68,69].

Mulberry-based food products are difficult to be labeled with DNJ contents since the quantification of DNJ through spectral analysis needs derivatization; in order to tackle this problem, HPAEC-PAD method was found useful. The HPAEC-PAD method was coupled with CarboPac MA1 column and sodium hydroxide gradient. As compared to high-performance liquid chromatography (HPLC), HPAEC-PAD was found to be more selective, simple, rapid, and sensitive method of DNJ analysis in mulberry-based food products in terms of good resolution (no interference of DNJ with other contents), simple sample preparation (water extract of mulberry tea sample), and time-consumption (retention time as low as 7.26 min), and sensitivity (limit of detection as low as 5 ng). This method was also found equally effective for mulberry-based products sterilized by heat treatment [7]. Moreover, most of the reported reversed-phase high performance liquid chromatography (HPLC) methods for DNJ determination based on pre-column derivatization involved the use of fluorescence detector, C 18 column, acetonitrile: 0.1% acetic acid (50:50, *v*/*v*) as mobile phase with a flow rate of 1.0 mL·min^−1^, and excitation and emission wavelengths 254 nm and 322 nm, respectively [7,53,70].

### 2.4. Chemistry

Polyhydroxylated piperidines and their derivatives are famous for their excellent bioactivities [1]. The iminosugars contain an analog of pyranose ring or d-glucose, in which the ring oxygen atom is replaced by a nitrogen atom. 1-Deoxyiminosugars are chemically more stable than normal iminosugars because of absence of a hydroxyl group at the C1 position. Among the iminosugars, naturally occurring 1-deoxyiminosugars such as DNJ are strong glycosidase inhibitors [2]. Originally, reduction of nojirimycin led to the chemical synthesis of DNJ [3]; afterward, DNJ was found from natural source, i.e., mulberry tree root [4]. These iminosugars, also known as azasugars, have attracted great attention of chemists and pharmacologists. Chemists have synthesized various DNJ derivatives, for instance *N*-hydroxyethyl-DNJ 7 (Miglitol) and *N*-butyl-DNJ 8 (Zavesca). Both are FDA approved drugs for non-insulin-dependent diabetes [5].

### 2.5. Oral Pharmacokinetic of 1-Deoxynojirimycin

The pharmacokinetic of DNJ after oral administration has been studies by some researchers [17,18,19]. It has been reported that there is a proportional increase in plasma DNJ level with increase in mulberry derived DNJ dose (1.1, 11, and 110 mg/kg of body weight), a dose-dependent phenomenon. An improved bioavailability was observed by pure DNJ versus the mulberry leaf extract administered to rats. It has been narrated that the plasma levels of mulberry derived DNJ after single oral administration (110 mg/kg of body weight) rapidly inclined reaching to a maximum level of 15 µg /mL, followed by quick decline in its level due to its rapid excretion from the body with a T_max_ (time to reach maximum plasma drug concentration) value of 30 min [17,18]. On the other hand, the T_max_ values of acarbose and miglitol were 1.27 h and 2.5 h, respectively [19,71]. It indicates that absorption and excretion of DNJ is faster than both acarbose and miglitol, which elaborates prolonged therapeutic effect of both acarbose and miglitol as compared to that of pure DNJ. The difference in absorption of acarbose, miglitol, and DNJ may be owing to their slight structural differences. The ethanol hydroxyl group of miglitol may modify the lipo-hydro partition coefficient. It leads to its reduced affinity for glucosyltransferase and glucose transporter, resulting in the slower absorption rate of miglitol as compared to that of DNJ. This information paved the path to development of a hypothesis, i.e., the formulation adjuvant may slow down the absorption rate of DNJ resulting in the improved postprandial hypoglycemic activity in vivo. In 2012, Wang et al. studied the influence of carboxymethylcellulose sodium (CMCNa) as an adjuvant on the absorption rate of DNJ. The results revealed that the absorption rate of DNJ decreased when DNJ and CMCNa were concomitantly ingested to rats through oral route. This change in pharmacokinetics of DNJ leads to an improved antihyperglycemic effect. Conclusively, CMCNa was found to be involved in modifying pharmacokinetics and pharmacodynamics of DNJ in rats [72]. In another pharmacokinetic study of DNJ, Xu et al. (2012) calculated the values of various pharmacokinetic parameters including area under plasma drug concentration curve (AUC), maximum plasma drug concentration (C_max_), T_max_, and K_a_ and found values were as here: 19.22 ± 1.37 mg·h/L, 12.98 ± 1.92 mg/L, 0.50 ± 0.10 h, and 4.85 ± 0.95 h^−1^, respectively. Moreover, DNJ is not metabolized in the rat plasma after oral administration [17]. Faber et al. have studied the distribution and elimination of DNJ in the rat after intravenous administration. Plasma protein binding of DNJ was very low. DNJ disappeared from plasma in two phases, with an initial and terminal half life of 3 min and 51 min, respectively. Major mode of DNJ elimination is the renal excretion. DNJ does not undergo tubular reabsorption. Bile and feces also contains some percentage of DNJ dose [73].

### 2.6. Biological Activities

1-Deoxynojirimycin has many biological activities, including antihyperglycemic [20,21,22,23,24,25], anti-obesity [26,27,28,29,30], and anti-viral [31] have also been reported [31,32,33,34,35]. (Table 1)

### 2.7. Antihyperglycemic Activity

**Figure 4:**
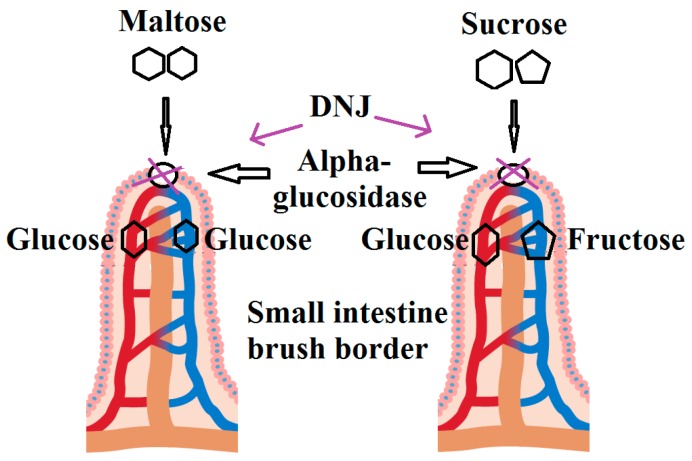
Supposed mode of DNJ action in the digestive tract.

Our diet consists of various essential components including the carbohydrates, for example sucrose, maltose, and starch. During the process of digestion, these are complex molecules, which undergo reactions under the effect of various enzymes [74]. For example, the pancreatic α-amylase plays key role in the conversion of starch into its oligosaccharides including maltose, isomaltose, maltotriose, and α-dextrins in duodenum and jejunum. Further, digestion of these hydrolytic products of starch into the monosaccharides is needed for their intestinal absorption [75]. This conversion is principally modulated by α-glucosidase enzymes, present in the brush-border membrane of the small intestine. Two important enzyme of this family are malto-glucoamylase and sucrose-isomaltase. These enzymes modulate the cleavage of α-1,4 linkages in oligosaccharides and α-1,6 linkages in α-dextrins, respectively [76]. d-glucose, the main product of the α-glucosidase-mediated hydrolysis, can be actively delivered across the mucosal membrane by glucosyltransferase and glucose transporter [77], leading to elevated level of blood glucose. Glucose transport across the intestinal membrane can be hindered by using α-glucosidase inhibitors, which can competitively bind to the catalytic site of α-glucosidase [78]. The important examples of α-glucosidase inhibitors are acarbose, miglitol, and DNJ; the former possess potential to bind to α-amylase, while α-glucosidase is a promising target site of the later two compounds [79,80,81]. The mode of action of DNJ involves the suppression of intestinal α-1,4-glucosidase as well as α-1,6-glucosidase of hepatic glycogen-debranching enzymes leading to the reduced rate of oligosaccharide breakdown [81]. The antihyperglycemic activity of DNJ has widely been studied [57]. DNJ is capable of binding to and inhibiting α-glycosidase and glucoamylase [22,23,24,25], leading to decrease in hepatic glucose metabolism and postprandial hyperglycemia [71]. Mechanistically, DNJ induces the inhibition of intestinal glucose absorption by diminishing the expression of proteins engaged in the transepithelial glucose transport (Figure 4). Moreover, the down-regulation of intestinal SGLT1, Na^+^/K^+^-ATP and GLUT2 mRNA and protein expression is also provoked by DNJ [82]. The anti-hyperglycemic effect of DNJ is evident from another study that DNJ plays a significant role in improving the insulin sensitivity through the activation of insulin signaling PI3K/AKT pathway in skeletal muscle of hyperglycemic model mice [53].

Adiponectin and its receptors in differentiated 3T3-L1 adipocytes have been found to be effective in reducing blood glucose levels and improving insulin sensitivity. In this view, there is an enhancement effect of DNJ (0.5 µM) on following parameters: (i) the levels of adiponectin and its receptors (AdipoR1 and AdipoR2) in differentiated 3T3-L1 adipocytes; (ii) phosphorylation of 5' adenosine monophosphate-activated protein kinase (AMPK); (iii) mRNA expression of glucose transporter 4 (GLUT4); and (iv) an excellent enhancement in glucose uptake into the adipocytes [83]. Consequently, strong anti-hyperglycemic effects of DNJ may be attained by large quantity of GLUT4 protein in the plasma membrane due to the enhanced transcript levels of the GLUT4 gene and the activation of AMPK. Thus, DNJ can be used to prevent or treat hyperglycemia.

### 2.8. Anti-Obesity Activity

The lipoprotein lipase-mediated conversion of serum VLDL (Very low density lipoprotein, known as very bad cholesterol) to LDL (low density lipoprotein) at a rate lower than normal leads to hypertriglyceridemia, the elevated VLDL level [84], which is a risk factor for arteriosclerosis. Arteriosclerosis is a side effect of lipid oxidation, while VLDL is more prone to oxidation. The hypertriglyceridemia can be prevented by mediating the continuous conversion of serum VLDL to LDL at normal rate. In an attempt to explore the effect of DNJ on TG level, DNJ-rich mulberry leaf extract (12 mg) was given to nine human subjects three times daily before meals over a period of 12 weeks. On Day 12, an elevated level of LDL but reduced contents of VLDL was observed [32]. It can be recommended that use of DNJ-rich mulberry leaf extract may be valuable by improving plasma lipoprotein profile.

Many tissues and organs in human body play a crucial role in maintaining normal metabolic balance. The metabolic imbalance can result in metabolic disorders, such as diabetes and obesity [85]. Adipose tissue is a loose connective tissue made majorly of adipocytes, which are originated from preadipocytes through a process known as adipogenesis. Adipose tissue not only acts a store-house for lipids, but also behaves as a major endocrine organ. This glandular organ produces hormones such as leptin, estrogen, and resistin. In short, adipose tissue is involved in metabolic regulation and prevention of harmful lipid accumulation in body [86]. It has been documented that DNJ prevents diet-provoked obesity via activation of β-oxidation system and augmented adiponectin levels, which inhibited lipid buildup in the liver and suppressed plasma triacylglycerol level [47]. Another study has stated that mulberry leaf ethanol extract (MLEE) treatment exerts anti-obesity through anti-adipogenic action in differentiated adipocytes [87]. From another study based on adipogenesis, it has been noted that 4 μM DNJ significantly suppresses adipogenesis. The possible mode of adipogenesis suppression by DNJ is extracellular regulated protein kinases 1/2/Peroxisome proliferator-activated receptor signaling pathway in the adipocytes [33]. Another study has reported the anti-obesity feature of DNJ. The investigators studied the influence of *Bacillus subtilis*-based DNJ on hepatic lipid metabolism and mitochondrial status of model mice (C57BL/6 mice) fed a fat-rich diet for twelve weeks. By Week 12, control (group of mice that received neither fat-rich diet nor DNJ) and DNJ (group of mice that received DNJ in addition to fat-rich diet) group mice did not show weight gain, unlike the HF group (group of mice that received fat-rich diet only), which showed significant weight gain. The hepatic C/EBPα and CD36 mRNA of the HF group also was highly expressed as compared to that of the control and DNJ group, which showed, on the other hand, the higher expression of hepatic p-AMPK/AMPK and PGC-1β mRNA [31]. These results give explanation for the proposed use of DNJ as a dietary supplement to avoid obesity and its consequences.

### 2.9. Anti-Viral Activity

The antiviral activity of DNJ has also been studied. There are several studies that describe the mode of action of DNJ against HIV [88,89,90,91,92]. One of the studies has reported that DNJ inhibits the spread of human immunodeficiency virus (HIV). They conducted this study virus-associated oligomeric Env [30]. Later on, the activity of silkworm extract and one of its purified constituents, DNJ, was compared against bovine viral diarrhea virus (BVDV), GB virus-B (GBV-B), woodchuck hepatitis virus (WHV), and hepatitis B virus (HBV). Against all these viruses, the effectiveness of the silkworm extract was significantly greater than that of purified DNJ. It can be assumed that five constituent iminosugars present in the silkworm extract contribute to the antiviral effect in a synergistic manner [93]. Additionally, Kang et al. studied the influence of DNJ (in a concentration of 10 mM DNJ) on the replication of Baculoviruses, *Bombyx Mori* Nucleopolyhedrovirus (BmNPV) and *Autographa Californica* Multiple Nucleopolyhedrovirus (AcMNPV). The results revealed that there was no effect of DNJ on the replication of Baculoviruses and BmNPV. However, the replication of AcMNPV was suppressed by 67%, this suppresses replication of AcMNPV was attributed to the higher sensitivity of α-glucosidase activity to DNJ [94]. It can be suggested that use of DNJ may be useful against viral infections, however further studies should be conducted to obtain benefit from this valuable phytochemical.

### 2.10. In Silico Target Fishing

After compiling all above data, we used PASS Prediction (Prediction of Activity Spectra for Substances) software to predict various types of biological activity potential of a small organic molecule on the basis of its structure, particularly before their chemical synthesis and biological analysis. The data required for predicting through PASS is “SMILES” or a structural formula of the compound in MOLfile format. Through this search tool, 44 possible activities of DNJ with a probability of Pa > 0.7 (probability to be active) were found (Table 2). It clearly indicates that DNJ can be used as anti-diabetic drug.

**Figure 5:**
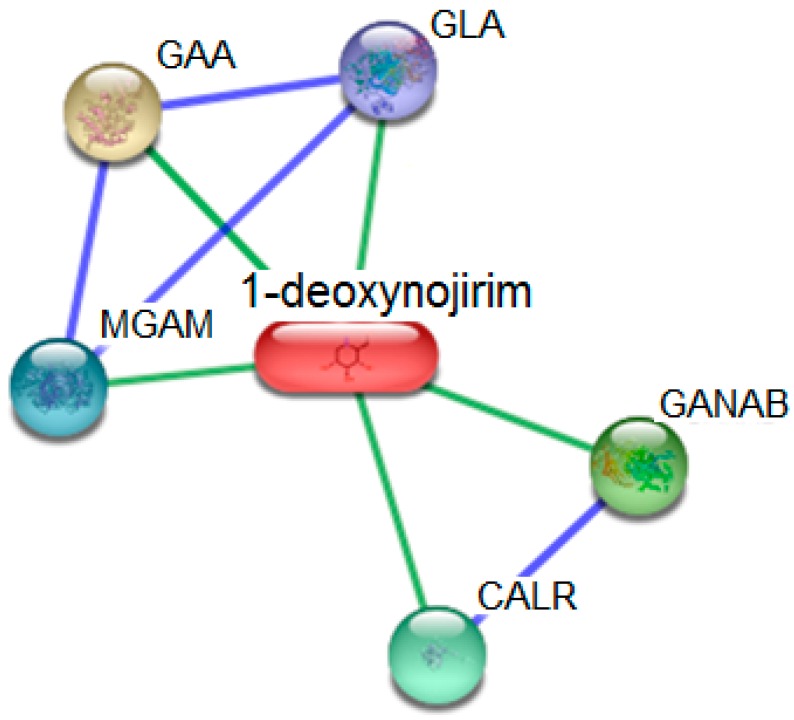
The protein network of DNJ. The proteins and their relationships are represented by the nodes and edges.

Subsequently, to narrow down the target search, we used STITCH for in silico target fishing in order to identify protein targets of DNJ as well as to predict the possible interactions of DNJ with other chemicals and proteins. STITCH, a database of protein interactions, integrates many sources of information, i.e., text mining, experimental evidences, and other databases including STRING. Targets with a confidence score > 0.7 were opted for construction of protein interaction network. Predicted DNJ targets and the probabilistic confidence score are presented in Table 3. The protein network of DNJ is shown Figure 5. In this confidence view, stronger interactions are represented by thicker lines (since all lines are thick, all the interactions are strong). Protein–protein interacting couples are CALR–GANAB, MGAM–GAA, MGAM–GLA, and GLA–GAA. Chemical (DNG)–protein associations are found between following couples: DNJ–CALR, DNJ–GANAB, DNJ–MGAM, DNJ–GLA, and DNJ–GAA. 

## 3. Conclusions

1-Deoxynojirimycin is biologically active with promising health effects. Its sources include mulberry leaves, *Commelina communis* (dayflower), and several bacterial strains such as *Bacillus* and *Streptomyces* species. Moreover, DNJ concentration is found to be different in different parts of mulberry tree. DNJ constitutes only 0.11% (*w*/*w*) of mulberry leaf. 1-Deoxynojirimycin possesses antihyperglycemic, anti-obesity, and antiviral features. Most importantly, pre-meal intake of DNJ in therapeutic concentration has resulted in the inhibition of postprandial hyperglycemia and hyperinsulinemia. Thus, DNJ seems to be a potential treatment for checking or setting back the inception of diabetes. No study is reported on toxicity of DNJ despite its long term use. Additional safety assessment on the pharmacokinetics, i.e., absorption, distribution, metabolism, and excretion of DNJ is needed prior to be utilized as a useful food. In silico target fishing has depicted 44 potential targets of DNJ in addition to α-glycosidase. However, further in silico and experimental validation is required to verify these findings, which may open new doors for drug development. In this regard, docking studies will first be carried in future to explore the binding mechanism of DNJ with these enzymes to elucidate the possible orientation and strength of binding affinity between DNG and recently identified target proteins. Following docking, MD simulation will be carried out to gain insight into the structural dynamics and stability of DNG in complex with these proteins.

## Figures and Tables

**Table 1:** Biological activities of 1-Deoxynojirimycin.

No.	Biological Activity	Reference
1	Antihyperglycemic	[20,21,22,23,24,25]
2	Anti-viral	[26,27,28,29,30]
3	Anti-obesity	[31,32,33,34,35]

**Table 2:** List of activities with a probability at Pa > 0.7 (probability to be active).

No.	Pa	Activity	No.	Pa	Activity
1	0.953	Phosphatidylcholine-sterol *O*-acyltransferase inhibitor	23	0.829	CDP-glycerol glycerophosphotransferase inhibitor
2	0.937	Glucan 1,3-β-glucosidase inhibitor	24	0.802	β-glucosidase inhibitor
3	0.924	l-iduronidase inhibitor	25	0.801	Fucosterol-epoxide lyase inhibitor
4	0.916	Mannosyl-oligosaccharide 1,2-α-mannosidase inhibitor	26	0.792	Ceramide glucosyltransferase inhibitor
5	0.905	Glycosylceramidase inhibitor	27	0.798	Glucan endo-1,6-β-glucosidase inhibitor
6	0.903	Oligo-1,6-glucosidase inhibitor	28	0.783	Mucinaminylserine mucinaminidase inhibitor
7	0.886	Sucrose α-glucosidase inhibitor	29	0.776	Fructan β-fructosidase inhibitor
8	0.880	Mannosidase inhibitor	30	0.773	Manganese peroxidase inhibitor
9	0.879	Glucan 1,3-α-glucosidase inhibitor	31	0.784	Alkenylglycerophosphocholine hydrolase inhibitor
10	0.880	β-mannosidase inhibitor	32	0.788	Testosterone 17β-dehydrogenase (NADP+) inhibitor
11	0.869	Sugar-phosphatase inhibitor	33	0.750	β-glucosidase inhibitor
12	0.856	α-mannosidase inhibitor	34	0.753	Glucan 1,4-α-maltotriohydrolase inhibitor
13	0.850	Mannosyl-oligosaccharide glucosidase inhibitor	35	0.755	Ribulose-phosphate 3-epimerase inhibitor
14	0.853	Nicotinic α6β3β4α5 receptor antagonist	36	0.741	α-glucosidase inhibitor
15	0.842	Amylo-α-1,6-glucosidase inhibitor	37	0.756	Benzoate-CoA ligase inhibitor
16	0.838	UDP-*N*-acetylglucosamine 4-epimerase inhibitor	38	0.730	Glucan 1,4-α-glucosidase inhibitor
17	0.833	α-l-fucosidase inhibitor	39	0.727	α,α-trehalose phosphorylase inhibitor
18	0.828	Exoribonuclease II inhibitor	40	0.721	Endo-1,3(4)-β-glucanase inhibitor
19	0.827	Nicotinic α2β2 receptor antagonist	41	0.708	Nucleoside oxidase (H_2_O_2_-forming) inhibitor
20	0.829	Glutamate-5-semialdehyde dehydrogenase inhibitor	42	0.717	Acylcarnitine hydrolase inhibitor
21	0.812	Interleukin 4 antagonist	43	0.704	Mannotetraose 2-α-*N*-acetylglucosaminyl transferase inhibitor
22	0.808	β-galactosidase inhibitor	44	0.723	Membrane integrity agonist

**Table 3:** Predicted DNJ targets.

Node Color	Abbreviations	Protein Targets	UniProt ID	Score
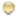	GLA	Galactosidase, α (429 aa)	P10253	0.921
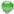	GANAB	Glucosidase, α; neutral AB; Cleaves sequentially the 2 innermost α-1,3-linked glucose residues from the Glc(2)Man(9)GlcNAc(2) oligosaccharide precursor of immature glycoproteins (966 aa)	Q14697	0.854
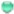	CALR	Calreticulin; Molecular calcium binding chaperone promoting folding, oligomeric assembly and quality control in the ER via the calreticulin/calnexin cycle. This lectin interacts transiently with almost all of the monoglucosylated glycoproteins that are synthesized in the ER. Interacts with the DNA-binding domain of NR3C1 and mediates its nuclear export (417 aa)	P27797	0.843
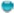	MGAM	Maltase-glucoamylase (α-glucosidase); May serve as an alternate pathway for starch digestion when luminal α-amylase activity is reduced because of immaturity or malnutrition. May play a unique role in the digestion of malted dietary oligosaccharides used in food manufacturing (1857 aa)	O43451	0.833
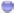	GAA	Glucosidase, α; acid; Essential for the degradation of glygogen to glucose in lysosomes (952 aa)	P06280	0.833

## References

[1] Pearson M.S.M., Mathe-Allainmat M., Fargeas V., Lebreton J. (2005). Recent Advances in the Total Synthesis of Piperidine Azasugars. Annalen Der Chemie Und Pharmacie.

[2] Zechel D.L., Withers S.G. (2000). Glycosidase mechanisms: Anatomy of a finely tuned catalyst. Acc. Chem. Res..

[3] Inouye S., Tsuruoka T., Ito T., Niida T. (1968). Structure and synthesis of nojirimycin. Tetrahedron.

[4] Yagi M., Kouno T., Aoyagi Y., Murai H. (1976). The structure of moranoline, a piperidine alkaloid from *Morus* species. J. Agric. Chem. Soc. Jpn..

[5] Butters T.D., Dwek R.A., Platt F.M. (2003). Therapeutic applications of imino sugars in lysosomal storage disorders. Curr. Top. Med. Chem..

[6] Vichasilp C., Nakagawa K., Sookwong P., Higuchi O., Luemunkong S., Miyazawa T. (2012). Development of high 1-deoxynojirimycin (DNJ) content mulberry tea and use of response surface methodology to optimize tea-making conditions for highest DNJ extraction. LWT Food Sci. Technol..

[7] Bajpai S., Rao A.V.B. (2014). Quantitative determination of 1-Deoxynojirimycin in different Mulberry Varieties of India. J. Pharm. Phytochem..

[8] Onose S., Ikeda R., Nakagawa K., Kimura T., Yamagishi K., Higuchi O., Miyazawa T. (2013). Production of the α-glycosidase inhibitor 1-deoxynojirimycin from Bacillus species. Food Chem..

[9] Ezure Y., Maruo S., Miyazaki K., Kawamata M. (1985). Moranoline (1-deoxynojirimycin) fermentation and its improvement. Agric. Biol. Chem..

[10] Sharpless K.E., Margolis S., Thomas J.B. (2000). Determination of vitamins in food-matrix Standard Reference Materials. J. Chromatogr. A.

[11] Kimura T., Nakagawa K., Saito Y., Yamagishi K., Suzuki M., Yamaki K., Shinmoto H., Miyazawa T. (2004). Determination of 1-deoxynojirimycin in mulberry leaves using hydrophilic interaction chromatography with evaporative light scattering detection. J. Agric. Food Chem..

[12] Yoshihashi T., Do H.T.T., Tungtrakul P., Boonbumrung S., Yamaki K. (2010). Simple, Selective, and Rapid Quantification of 1-Deoxynojirimycin in Mulberry Leaf Products by High-Performance Anion-Exchange Chromatography with Pulsed Amperometric Detection. J. Food Sci..

[13] Einarsson S., Josefsson B., Lagerkvist S. (1983). Determination of amino acids with 9-fluorenylmethyl chloroformate and reversed-phase high-performance liquid chromatography. J. Chromatogr. A.

[14] Lewis J., Morley J., Venn R. (1993). Analysis of human β-endorphin 28–31 (melanotropin potentiating factor) and analogues by high-performance liquid chromatography of their 9-fluorenylmethoxy-carbonyl derivatives. J. Chromatogr. B Biomed. Sci. Appl..

[15] Stead D., Richards R. (1996). Sensitive fluorimetric determination of gentamicin sulfate in biological matrices using solid-phase extraction, pre-column derivatization with 9-fluorenylmethyl chloroformate and reversed-phase high-performance liquid chromatography. J. Chromatogr. B Biomed. Sci. Appl..

[16] Shangguan D., Zhao Y., Han H., Zhao R., Liu G. (2001). Derivatization and fluorescence detection of amino acids and peptides with 9-fluorenylmethyl chloroformate on the surface of a solid adsorbent. Anal. Chem..

[17] Nakagawa K., Kubota H., Kimura T., Yamashita S., Tsuzuki T., Oikawa S., Miyazawa T. (2007). Occurrence of orally administered mulberry 1-deoxynojirimycin in rat plasma. J. Agric. Food Chem..

[18] Kim J.Y., Kwon H.J., Jung J.Y., Kwon H.Y., Baek J.G., Kim Y.-S., Kwon O. (2010). Comparison of absorption of 1-deoxynojirimycin from mulberry water extract in rats. J. Agric. Food Chem..

[19] Ahr H., Boberg M., Krause H., Maul W., Müller F., Ploschke H., Weber H., Wünsche C. (1989). Pharmacokinetics of acarbose. Part I: Absorption, concentration in plasma, metabolism and excretion after single administration of [14C] acarbose to rats, dogs and man. Arzneimittel-Forschung.

[20] Asano N., Yamashita T., Yasuda K., Ikeda K., Kizu H., Kameda Y., Kato A., Nash R.J., Lee H.S., Ryu K.S. (2001). Polyhydroxylated alkaloids isolated from mulberry trees (*Morus alba* L.) and silkworms (*Bombyx mori* L.). J. Agric. Food Chem..

[21] Gui Z., Zhuang D., Chen J., Chen W. (2000). Effect of silkworm powder (SP) lowering blood-glucose levels in mice and its mechanism. Acta Sericol. Sin..

[22] Bembi B., Deegan P. (2008). Gaucher disease: Improving management. Acta Paediatr..

[23] Kuriyama C., Kamiyama O., Ikeda K., Sanae F., Kato A., Adachi I., Imahori T., Takahata H., Okamoto T., Asano N. (2008). In vitro inhibition of glycogen-degrading enzymes and glycosidases by six-membered sugar mimics and their evaluation in cell cultures. Bioorg. Med. Chem..

[24] Newbrun E., Hoover C., Walker G.J. (1983). Inhibition by acarbose, nojirimycin and 1-deoxynojirimycin of glucosyltransferase produced by oral streptococci. Arch. Oral Biol..

[25] Yatsunami K., Ichida M., Onodera S. (2008). The relationship between 1-deoxynojirimycin content and α-glucosidase inhibitory activity in leaves of 276 mulberry cultivars (*Morus* spp.) in Kyoto, Japan. J. Nat. Med..

[26] Chang J., Wang L., Ma D., Qu X., Guo H., Xu X., Mason P.M., Bourne N., Moriarty R., Gu B. (2009). Novel imino sugar derivatives demonstrate potent antiviral activity against flaviviruses. Antimicrob. Agents Chemother..

[27] Tanaka Y., Kato J., Kohara M., Galinski M.S. (2006). Antiviral effects of glycosylation and glucose trimming inhibitors on human parainfluenza virus type 3. Antivir. Res..

[28] Durantel D., Branza-Nichita N., Carrouée-Durantel S., Butters T.D., Dwek R.A., Zitzmann N. (2001). Study of the mechanism of antiviral action of iminosugar derivatives against bovine viral diarrhea virus. J. Virol..

[29] Lazar C., Durantel D., Macovei A., Zitzmann N., Zoulim F., Dwek R.A., Branza-Nichita N. (2007). Treatment of hepatitis B virus-infected cells with α-glucosidase inhibitors results in production of virions with altered molecular composition and infectivity. Antivir. Res..

[30] Papandréou M.-J., Barbouche R., Guieu R., Kieny M.P., Fenouillet E. (2002). The α-glucosidase inhibitor 1-deoxynojirimycin blocks human immunodeficiency virus envelope glycoprotein-mediated membrane fusion at the CXCR4 binding step. Mol. Pharmacol..

[31] Do H.J., Chung J.H., Hwang J.W., Kim O.Y., Lee J.-Y., Shin M.-J. (2015). 1-Deoxynojirimycin isolated from Bacillus subtilis improves hepatic lipid metabolism and mitochondrial function in high-fat–fed mice. Food Chem. Toxicol..

[32] Kojima Y., Kimura T., Nakagawa K., Asai A., Hasumi K., Oikawa S., Miyazawa T. (2010). Effects of mulberry leaf extract rich in 1-deoxynojirimycin on blood lipid profiles in humans. J. Clin. Biochem. Nutr..

[33] Wang G.-Q., Zhu L., Ma M.-L., Chen X.-C., Gao Y., Yu T.-Y., Yang G.-S., Pang W.-J. (2015). Mulberry 1-Deoxynojirimycin Inhibits Adipogenesis by Repression of the ERK/PPARγ Signaling Pathway in Porcine Intramuscular Adipocytes. J. Agric. Food Chem..

[34] Kong W.-H., Oh S.-H., Ahn Y.-R., Kim K.-W., Kim J.-H., Seo S.-W. (2008). Antiobesity effects and improvement of insulin sensitivity by 1-deoxynojirimycin in animal models. J. Agric. Food Chem..

[35] Monte S.V., Schentag J.J., Adelman M.H., Paladino J.A. (2010). Glucose supply and insulin demand dynamics of antidiabetic agents. J. Diabetes sci. Technol..

[36] Koutsoukas A., Simms B., Kirchmair J., Bond P.J., Whitmore A.V., Zimmer S., Young M.P., Jenkins J.L., Glick M., Glen R.C. (2011). From in silico target prediction to multi-target drug design: Current databases, methods and applications. J. Proteom..

[37] Jenkins J.L., Bender A., Davies J.W. (2007). In silico target fishing: Predicting biological targets from chemical structure. Drug Discov. Today Technol..

[38] Chen F., Nakashima N., Kimura I., Kimura M. (1995). [Hypoglycemic activity and mechanisms of extracts from mulberry leaves (*Folium mori*) and cortex mori radicis in streptozotocin-induced diabetic mice]. Yakugaku Zasshi J. Pharm. Soc. Jpn..

[39] Hocking D. (1993). Trees For Drylands.

[40] Arabshahi-Delouee S., Urooj A. (2007). Antioxidant properties of various solvent extracts of mulberry (*Morus indica* L.) leaves. Food Chem..

[41] Ercisli S. (2004). A short review of the fruit germplasm resources of Turkey. Genet. Resour. Crop Evol..

[42] Halliwell B. (2007). Dietary polyphenols: Good, bad, or indifferent for your health?. Cardiovasc. Res..

[43] Manach C., Mazur A., Scalbert A. (2005). Polyphenols and prevention of cardiovascular diseases. Curr. Opin. Lipidol..

[44] Ercisli S., Orhan E. (2007). Chemical composition of white (*Morus alba*), red (*Morus rubra*) and black (*Morus nigra*) mulberry fruits. Food Chem..

[45] Butt M.S., Nazir A., Sultan M.T., Schroën K. (2008). Morus alba L. nature's functional tonic. Trends Food Sci. Technol..

[46] Kooij R., Branderhorst H.M., Bonte S., Wieclawska S., Martin N.I., Pieters R.J. (2013). Glycosidase inhibition by novel guanidinium and urea iminosugar derivatives. MedChemComm.

[47] Tsuduki T., Kikuchi I., Kimura T., Nakagawa K., Miyazawa T. (2013). Intake of mulberry 1-deoxynojirimycin prevents diet-induced obesity through increases in adiponectin in mice. Food Chem..

[48] Kimuar M., Chen F., Nakashima N. (1995). Antihyperglycemic effects of N-containing sugars derived from mulberry leaves is streptozocin-in-duced diabetic mice. Wakan Iyakugaku Zasshi.

[49] Lee S.H., Choi S.Y., Kim H., Hwang J.S., Lee B.G., Gao J.J., Kim S.Y. (2002). Mulberroside F isolated from the leaves of Morus alba inhibits melanin biosynthesis. Biol. Pharm. Bull..

[50] Fang S.-H., Hou Y.-C., Chao P.-D.L. (2005). Pharmacokinetic and pharmacodynamic interactions of morin and cyclosporin. Toxicol. Appl. Pharmacol..

[51] Niidome T., Takahashi K., Goto Y., Goh S., Tanaka N., Kamei K., Ichida M., Hara S., Akaike A., Kihara T. (2007). Mulberry leaf extract prevents amyloid β-peptide fibril formation and neurotoxicity. Neuroreport.

[52] Jiang Y.-G., Wang C.-Y., Jin C., Jia J.-Q., Guo X., Zhang G.-Z., Gui Z.-Z. (2014). Improved 1-Deoxynojirimycin (DNJ) production in mulberry leaves fermented by microorganism. Braz. J. Microbiol..

[53] Liu Q., Li X., Li C., Zheng Y., Peng G. (2015). 1-Deoxynojirimycin Alleviates Insulin Resistance via Activation of Insulin Signaling PI3K/AKT Pathway in Skeletal Muscle of db/db Mice. Molecules.

[54] Asai A., Nakagawa K., Higuchi O., Kimura T., Kojima Y., Kariya J., Miyazawa T., Oikawa S. (2011). Effect of mulberry leaf extract with enriched 1-deoxynojirimycin content on postprandial glycemic control in subjects with impaired glucose metabolism. J. Diabetes Investig..

[55] Schmidt D., Frommer W., Junge B., Müller L., Wingender W., Truscheit E., Schäfer D. (1977). α-Glucosidase inhibitors. Naturwissenschaften.

[56] Kanieda Y., Asano N., Teranishi M., Matsui K. (1980). New cyclitols, degradation of validamycin a by Flavobacterium saccharophilum. J. Antibiot..

[57] Kimura T., Nakagawa K., Kubota H., Kojima Y., Goto Y., Yamagishi K., Oita S., Oikawa S., Miyazawa T. (2007). Food-grade mulberry powder enriched with 1-deoxynojirimycin suppresses the elevation of postprandial blood glucose in humans. J. Agric. Food Chem..

[58] Song W., Wang H.-J., Bucheli P., Zhang P.-F., Wei D.-Z., Lu Y.-H. (2009). Phytochemical profiles of different mulberry (*Morus* sp.) species from China. J. Agric. Food Chem..

[59] Hu K., Li Y., Du Y., Su B., Lu D. (2011). Analysis of 1-deoxynojirimycin component correlation between medicinal parasitic loranthus from loranthaceae and their mulberry host trees. J. Med. Plants Res..

[60] Hu X.-Q., Jiang L., Zhang J.-G., Deng W., Wang H.-L., Wei Z.-J. (2013). Quantitative determination of 1-deoxynojirimycin in mulberry leaves from 132 varieties. Ind. Crop. Prod..

[61] Jeong J.H., Lee N.K., Cho S.H., Jeong Y.-S. (2014). Enhancement of 1-deoxynojirimycin content and α-glucosidase inhibitory activity in mulberry leaf using various fermenting microorganisms isolated from Korean traditional fermented food. Biotechnol. Bioprocess Eng..

[62] Wang T., Li C.-Q., Zhang H., Li J.-W. (2014). Response surface optimized extraction of 1-deoxynojirimycin from mulberry leaves (*Morus alba* L.) and preparative separation with resins. Molecules.

[63] Vichasilp C., Nakagawa K., Sookwong P., Suzuki Y., Kimura F., Higuchi O., Miyazawa T. (2009). Optimization of 1-deoxynojirimycin extraction from mulberry leaves by using response surface methodology. Biosci. Biotechnol. Biochem..

[64] Tolstikov V.V., Fiehn O. (2002). Analysis of highly polar compounds of plant origin: Combination of hydrophilic interaction chromatography and electrospray ion trap mass spectrometry. Anal. Biochem..

[65] Kim J.-W., Kim S.-U., Lee H.S., Kim I., Ahn M.Y., Ryu K.S. (2003). Determination of 1-deoxynojirimycin in *Morus alba* L. leaves by derivatization with 9-fluorenylmethyl chloroformate followed by reversed-phase high-performance liquid chromatography. J. Chromatogr. A.

[66] Nuengchamnong N., Ingkaninan K., Kaewruang W., Wongareonwanakij S., Hongthongdaeng B. (2007). Quantitative determination of 1-deoxynojirimycin in mulberry leaves using liquid chromatography–tandem mass spectrometry. J. Pharm. Biomed. Anal..

[67] Keiner R., Dräger B. (2000). Calystegine distribution in potato (*Solanum tuberosum*) tubers and plants. Plant Sci..

[68] Nakagawa K., Ogawa K., Higuchi O., Kimura T., Miyazawa T., Hori M. (2010). Determination of iminosugars in mulberry leaves and silkworms using hydrophilic interaction chromatography–tandem mass spectrometry. Anal. Biochem..

[69] Herraez-Hernandez R., Campins-Falco P. (2000). Derivatization of tertiary amphetamines with 9-fluorenylmethyl chloroformate for liquid chromatography: Determination of *N*-methylephedrine. Analyst.

[70] Feng Z., Zhe S., Chu Q., Lin T., Xin L., Feng X., Li Z., Yang S., Xianghui L., Jingxian Z. (2012). Therapeutic target database update 2012: A resource for facilitating target-oriented drug discovery. Nucleic Acids Res..

[71] Wang L., Peng J., Wang X., Zhu X., Cheng B., Gao J., Jiang M., Bai G., Hou Y. (2012). Carboxymethylcellulose sodium improves the pharmacodynamics of 1-deoxynojirimycin by changing its absorption characteristics and pharmacokinetics in rats. Pharm. Int. J. Pharm. Sci..

[72] Nirogi R.V., Kandikere V.N., Shukla M., Mudigonda K., Maurya S., Boosi R., Yerramilli A. (2006). Liquid chromatographic tandem mass spectrometry method for the quantification of miglitol in human plasma. Arzneimittelforschung.

[73] Faber E.D., Oosting R., Neefjes J.J., Ploegh H.L., Meijer D.K. (1992). Distribution and elimination of the glycosidase inhibitors 1-deoxymannojirimycin and *N*-methyl-1-deoxynojirimycin in the rat in vivo. Pharm. Res..

[74] Dyer J., Merediz E.F.-C., Salmon K., Proudman C., Edwards G., Shirazi-Beechey S. (2002). Molecular characterisation of carbohydrate digestion and absorption in equine small intestine. Equine Vet. J..

[75] Gray G.M. (1992). Starch digestion and absorption in nonruminants. J. Nutr..

[76] Koh L.W., Wong L.L., Loo Y.Y., Kasapis S., Huang D. (2009). Evaluation of different teas against starch digestibility by mammalian glycosidases. J. Agric. Food Chem..

[77] Drozdowski L., Thomson A.B. (2006). Citation of This Article. World J. Gastroenterol..

[78] Bischoff H. (1995). The mechanism of α-glucosidase inhibition in the management of diabetes. Clin. Investig. Med..

[79] Krentz A.J. (2006). Comparative safety of newer oral antidiabetic drugs. Expert Opin. Drug Saf..

[80] Sels J.-P.J., Huijberts M.S., Wolffenbuttel B.H. (1999). Miglitol, a new α-glucosidase inhibitor. Expert Opin. Pharmacother..

[81] Martin O. (2007). Iminosugars: Current and future therapeutic applications. Ann. Pharm. Francaises.

[82] Li Y.-G., Ji D.-F., Zhong S., Lin T.-B., Lv Z.-Q., Hu G.-Y., Wang X. (2013). 1-deoxynojirimycin inhibits glucose absorption and accelerates glucose metabolism in streptozotocin-induced diabetic mice. Sci. Rep..

[83] Lee S.M., Shin M.J., Hwang K.Y., Kwon O., Chung J.H. (2013). 1-Deoxynojirimycin isolated from a Bacillus subtilis stimulates adiponectin and GLUT4 expressions in 3T3-L1 adipocytes. J. Microbiol. Biotechnol..

[84] Oikawa T., Mukoyama S., Soda K. (2001). Chemo-enzymatic d-enantiomerization of dl-lactate. Biotechnol. Bioeng..

[85] Farmer S., Auwerx J. (2004). Adipose tissue: New therapeutic targets from molecular and genetic studies–IASO Stock Conference 2003 report. Obes. Rev..

[86] Kersten S. (2001). Mechanisms of nutritional and hormonal regulation of lipogenesis. EMBO Rep..

[87] Yang S.J., Park N.-Y., Lim Y. (2014). Anti-adipogenic effect of mulberry leaf ethanol extract in 3T3-L1 adipocytes. Nutr. Res. Pract..

[88] Dedera D., Vander Heyden N., Ratner L. (1990). Attenuation of HIV-1 infectivity by an inhibitor of oligosaccharide processing. AIDS Res. Hum. Retrovir..

[89] Fenouillet E., Gluckman J.-C. (1991). Effect of a glucosidase inhibitor on the bioactivity and immunoreactivity of human immunodeficiency virus type 1 envelope glycoprotein. J. Gen. Virol..

[90] Jones I.M., Jacob G.S. (1991). Anti-HIV drug mechanism. Nature.

[91] Ratner L. (1992). Glucosidase inhibitors for treatment of HIV-1 infection. AIDS Res. Hum. Retrovir..

[92] Fischer P., Collin M., Karlsson G., James W., Butters T., Davis S., Gordon S., Dwek R., Platt F. (1995). The α-glucosidase inhibitor *N*-butyldeoxynojirimycin inhibits human immunodeficiency virus entry at the level of post-CD4 binding. J. Virol..

[93] Jacob J.R., Mansfield K., You J.E., Tennant B.C., Kim Y.H. (2007). Natural iminosugar derivatives of 1-deoxynojirimycin inhibit glycosylation of hepatitis viral envelope proteins. J. Microbiol..

[94] Kang K.-D., Park J.-S., Cho Y.-S., Park Y.-S., Lee J.-Y., Hwang K.-Y., Yuk W.-J., Kamita S.G., Suzuki K., Seong S.-I. (2011). Effect of 1-deoxynojirimycin on the replication of baculoviruses, Bombyx mori Nucleopolyhedrovirus and Autographa californica multiple nucleopolyhedrovirus. Int. J. Ind. Entomol..

